# Efficacy and safety of immune checkpoint inhibitors and targeted therapies in resected melanoma: a systematic review and network meta-analysis

**DOI:** 10.3389/fphar.2023.1284240

**Published:** 2023-11-07

**Authors:** Feng Sheng, Yulan Yan, Baoqi Zeng

**Affiliations:** ^1^ Department of Dermatology, Peking University Binhai Hospital, Tianjin, China; ^2^ Hematology and Oncology, Peking University Binhai Hospital, Tianjin, China; ^3^ Central Laboratory, Peking University Binhai Hospital, Tianjin, China; ^4^ Department of Epidemiology and Biostatistics, School of Public Health, Peking University Health Science Centre, Beijing, China

**Keywords:** melanoma, immune checkpoint inhibitors, network meta-analysis, adjuvant treatment, efficacy

## Abstract

**Background:** Multiple immune checkpoint inhibitors (ICIs) and targeted therapies have been widely used as adjuvant treatments for high-risk resected melanoma, with unclear comparative efficacy and safety.

**Methods:** PubMed, Embase, the Cochrane Library, and ClinicalTrials.gov were searched from database inception until 6 June 2023. We included RCTs that assess adjuvant ICIs or targeted therapies in high-risk resected melanoma. Frequentist random-effect network meta-analyses (NMA) were performed. The primary outcome was recurrence-free survival (RFS).

**Results:** Eleven trials including 10,712 patients and comparing 10 treatments (nivolumab [Nivo], ipilimumab 3 mg/kg [Ipi3], Ipi10, pembrolizumab [Pemb], vemurafenib [Vemu], bevacizumab [Beva], Nivo + Ipi1, Nivo + Ipi3, dabrafenib plus trametinib [Dab + Tram], and placebo/observation [Pla/Obs]) were included. NMA showed that all treatments showed RFS benefit over placebo/observation except Ipi3 (hazard ratio [HR], 0.78; 95% CI, 0.58–1.05). Combination therapy of Nivo + Ipi3 was the most effective treatment, which significantly improved RFS compared with other treatments. NMA also showed that all treatments were associated with an increased risk of grade 3-5 adverse events over placebo/observation except Nivo (HR, 1.25; 95% CI, 0.87–1.80). NMA suggested that Nivo and Pemb were the two safest treatments except for placebo/observation. Although three combination therapies ranked as the top three in terms of RFS, they did not show significant overall survival benefits compared to monotherapies including Pemb, Nivo, Ipi3, and Ipi10.

**Conclusion:** In this NMA, adjuvant Nivo and Pemb are the preferred options in patients with resected melanoma considering the benefits and harms. Combination therapy of Nivo + Ipi3 may be a promising strategy, but more evidence from phase 3 trials is needed.

**Systematic Review Registration:** https://www.crd.york.ac.uk/PROSPERO/display_record.php?RecordID=438667, PROSPERO (CRD42023438667).

## 1 Introduction

Melanoma is a major public health problem and is the leading cause of skin cancer death worldwide, and has steadily increased in recent years (Stege et al., 2021; Siegel et al., 2023). It is estimated that 97,610 new cases of melanoma and 7,990 deaths will occur in 2023 in the United States (Siegel et al., 2023). Surgical excision is the preferred treatment for early-stage melanoma, yet 40%–60% of patients with high-risk melanoma or nodal involvement will eventually experience loco-regional relapse or tumor progression (Stege et al., 2021). Immune Checkpoint inhibitors (ICIs) and targeted therapy were approved for the treatment of melanoma in the United States and Europe. Several therapies have been shown to improve outcomes of patients with unresectable metastatic melanoma when used as the initial treatment, including ipilimumab, nivolumab, pembrolizumab, and combinations of BRAF plus MEK inhibitors (dabrafenib plus trametinib) in randomized control trials (RCTs). (Hodi et al., 2010; Long et al., 2014; Robert et al., 2015; Hodi et al., 2016; Larkin et al., 2019). In recent years, these treatments have been employed in the adjuvant setting to prevent the recurrence of melanoma following surgical excision (Long et al., 2017; Eggermont et al., 2018a). BRIM8 was a phase 3 trial that compared adjuvant vemurafenib to placebo, which demonstrated a potential improvement in disease-free survival (DFS) and well-tolerated treatment for patients with resected BRAF mutation-positive melanoma (Maio et al., 2018) Bevacizumab, a monoclonal antibody that targets VEGF, has shown improvement in DFS, but not in overall survival (OS) for patients with high-risk resected melanoma (Corrie et al., 2018). However, the optimal treatment regimen for high-risk resected melanoma remains unclear. Thus, we systematically reviewed evidence from RCTs to evaluate the efficacy and safety of different ICIs and targeted therapies for resected melanoma.

## 2 Materials and methods

### 2.1 Data sources and searches

We searched for literature published on PubMed, Embase, Cochrane Library, and grey literature from the ClinicalTrials.gov website up to date 6 June 2023. The search strategies were developed and piloted by the review team for bibliographic databases and clinical trial registries using medical subject headings or Emtree and text words for “adjuvant”, “resect”, melanoma”, and “randomized controlled trials” (see Supplementary Material). We conducted this meta-analysis according to the Preferred Reporting Items for Systematic Reviews and Meta-Analyses (PRISMA) guidelines, (Moher et al., 2009), and the protocol was registered on PROSPERO (CRD42023438667).

### 2.2 Eligibility criteria

We included RCTs (phase 2/3) that assessed adjuvant ICIs or targeted therapies in high-risk (American Joint Committee on Cancer stage IIB-IV) resected melanoma. Patients diagnosed with stage IIB and IIC melanoma, even in the absence of identified lymph node involvement, face a higher risk of recurrence and melanoma-specific death compared to those with stage IIIA disease and a similar risk to those with stage IIIB disease. (Yushak et al., 2019). Our search was restricted to papers published in English. We excluded conference abstracts, ongoing trials, and studies that had insufficient data to analyze. Two reviewers independently screened the titles and abstracts of all identified records. We retrieved full-length records of those deemed eligible and screened these again to confirm inclusion. Disagreements were resolved by discussion, or with the help of a third reviewer when consensus could not be reached.

### 2.3 Outcomes

The primary outcome was RFS. Secondary outcomes were distant metastasis-free survival (DMFS), overall survival (OS), and grade 3-5 adverse events (AEs). In some studies, only disease-free survival or relapse-free survival was reported, which were considered equivalent to recurrence-free survival.

### 2.4 Data extraction and quality assessment

Two reviewers independently extracted data on the study characteristics, patient characteristics, interventions, comparisons, and outcomes from each study using a standardized, piloted form. Hazard ratios (HRs) with 95% Cis were extracted for survival data if available. When studies presented outcomes for various follow-up periods, we extracted data for the longest follow-up period. Both ipilimumab at 3 mg/kg and 10 mg/kg were approved therapies for melanoma, we separated them as two treatment regimens. Since the combination therapy of nivolumab plus ipilimumab 1 mg/kg once every 6 weeks probably has different outcomes compared to nivolumab plus ipilimumab 3 mg/kg once every 3 weeks, (Weber et al., 2023), the two combination therapies were analysed separately. The risk of bias of RCTs was assessed by the Cochrane Collaboration’s tool (RoB 2), (Sterne et al., 2019), which includes the following domains: randomization process, deviations from intended interventions, missing outcome data, measurement of the outcome, and selection of the reported result.

## 3 Results

### 3.1 Characteristics of included studies

This systematic literature search initially identified 6,707 records, after excluding duplicates and irrelevant papers, 130 papers were evaluated in full text for eligibility (Supplementary Figure S1). Finally, 23 research papers originating from 11 RCTs enrolling 10,712 participants were included in this NMA. (Corrie et al., 2014; Eggermont et al., 2015; Eggermont et al., 2016; Long et al., 2017; Weber et al., 2017; Eggermont et al., 2018b; Corrie et al., 2018; Hauschild et al., 2018; Maio et al., 2018; Ascierto et al., 2020a; Ascierto et al., 2020b; Dummer et al., 2020; Eggermont et al., 2020; Tarhini et al., 2020; Zimmer et al., 2020; Eggermont et al., 2021; Grossmann et al., 2022; Larkin et al., 2022; Long et al., 2022; Luke et al., 2022; Zimmer et al., 2022; Larkin et al., 2023; Weber et al., 2023). Ten treatment strategies were analyzed, including nivolumab [Nivo], ipilimumab 3 mg/kg [Ipi3], Ipi10, pembrolizumab [Pemb], vemurafenib [Vemu], bevacizumab [Beva], Nivo + Ipi1, Nivo + Ipi3, dabrafenib plus trametinib [Dab + Tram], and placebo/observation [Pla/Obs]. The follow-up periods ranged from 3 to 5 years. Only one RCT was a phase 2 trial, (Zimmer et al., 2020), and the rest of included RCTs were phase 3 trials. One trial had observation as a control group compared with Beva. (Corrie et al., 2014). Five two-arm trials had placebo as a control group, with one compared with Vemu, (Maio et al., 2018), two compared with Pemb, (Eggermont et al., 2018b; Luke et al., 2022), one compared with Ipi10,^28^ and one compared with Dab + Tram. (Long et al., 2022). Three trials had Ipi10 as the control group compared with Nivo, Pemb, and Ipi3, respectively (Weber et al., 2017; Tarhini et al., 2020; Grossmann et al., 2022). One three-arm trial compared Nivo + Ipi3 with Nivo and placebo was included. (Zimmer et al., 2020). The remaining trial compared combination therapy of Nivo + Ipi1 with Nivo. (Weber et al., 2023). All trials were previously registered and provided registration numbers. Participants’ median age and proportion of men at baseline ranged from 50 to 61 years and from 45% to 62%, respectively. Characteristics of individual studies are summarised in Table 1.

**TABLE 1:** Summary of included randomized controlled trials.

Study, phase	Registration	AJCC stage	Patient characteristics	BRAF status	Treatments	No of patients	Follow-up
CheckMate 238, phase 3 (Weber et al., 2017; Ascierto et al., 2020b; Larkin et al., 2022; Larkin et al., 2023)	NCT02388906, and EudraCT, 2014-002351-26	stage IIIB–C and IV	Median (IQR) age 55 (44, 65), male (58%)	Mutant (43%), wild type (45%), not reported (12%)	Treatment 1: nivolumab 3 mg/kg every 2 weeks; Treatment 2: ipilimumab 10 mg/kg every 3 weeks	906	5 years
BRIM8, phase 3 (Maio et al., 2018; Ascierto et al., 2020a)	NCT01667419	stage IIC–IIIC	Median (IQR) age 50 (40, 60), male (57%)	Mutant (100%)	Treatment 1: vemurafenib 960 mg twice daily; Treatment 2: placebo	498	4 years
EORTC 1325/KEYNOTE-054, phase 3 (Eggermont et al., 2018b; Eggermont et al., 2020; Eggermont et al., 2021)	NCT02362594, and EudraCT, 2014-004944-37	stage III	Median (range) age 54 (19–88), male (62%)	Mutant (41%), wild type (52%), not reported (7%)	Treatment 1: pembrolizumab 200 mg every 3 weeks; Treatment 2: placebo	1,019	3.5 years
EORTC 18071, phase 3 (Eggermont et al., 2015; Eggermont et al., 2016)	NCT00636168, and EudraCT, 2007-001974-10	stage III	Median (range) age 52 (18–84), male (62%)	Not reported (100%)	Treatment 1: ipilimumab 10 mg/kg every 3 weeks; Treatment 2: placebo	951	5 years
AVAST-M, phase 3 (Corrie et al., 2014; Corrie et al., 2018)	ISRCTN81261306	stage IIB, IIC, and III	Median (range) age 55 (19–88), male (56%)	Mutant (19%), wild type (26%), not reported (55%)	Treatment 1: bevacizumab 7.5 mg/kg every 3 weeks; Treatment 2: observation	1,343	5 years
COMBI-AD, phase 3 (Long et al., 2017; Hauschild et al., 2018; Dummer et al., 2020)	NCT01682083, and EudraCT, 2012-001266-15	stage III	Median (range) age 50 (18–89), male (45%)	Mutant (100%)	Treatment 1: dabrafenib 150 mg twice daily plus trametinib 2 mg once daily; Treatment 2: placebo	870	5 years
KEYNOTE-716, phase 3 (Long et al., 2022; Luke et al., 2022)	NCT03553836	stage IIB or IIC	Median (IQR) age 61 (51, 69), male (60%)	Not reported (100%)	Treatment 1: pembrolizumab 200 mg every 3 weeks; Treatment 2: placebo	976	3 years
SWOG S1404, phase 3 (Grossmann et al., 2022)	NCT02506153	stage III and IV	Median (range) age 54 (18–86), male (60%)	Mutant (22%), wild type (26%), not reported (52%)	Treatment 1: pembrolizumab 200 mg every 3 weeks; Treatment 2: ipilimumab 10 mg/kg every 3 weeks	1,115	5 years
IMMUNED, phase 2 (Zimmer et al., 2020; Zimmer et al., 2022)	NCT02523313	stage IV	Median (IQR) age 55 (46, 65), male (57%)	Mutant (45%), wild type (55%)	Treatment 1: nivolumab 3 mg/kg every 2 weeks plus ipilimumab 3 mg/kg every 3 weeks; Treatment 2: nivolumab 3 mg/kg every 2 weeks; Treatment 3: placebo	167	4 years
CheckMate 915, phase 3 (Weber et al., 2023)	NCT03068455	Stage IIIB-D or IV	Median (range) age 55 (15–89), male (57%)	Mutant (31%), wild type (46%), not reported (23%)	Treatment 1: nivolumab 240 mg once every 2 weeks plus ipilimumab 1 mg/kg once every 6 weeks; Treatment 2: nivolumab 480 mg once every 4 weeks	1833	3 years
E1609, phase 3 (Tarhini et al., 2020)	NCT01274338	stage IIIB, IIIC, or IV (M1a or M1b)	Median (range) age 54 (18–83), male (57%)	Not reported (100%)	Treatment 1: ipilimumab 10 mg/kg; Treatment 2: ipilimumab 3 mg/kg	1,034	5 years

IQR, interquartile range; AJCC, american joint committee on cancer.

### 3.2 Risk of bias

A qualitative assessment was performed by assessing various indicators for each individual trial using RoB 2. Eight of 11 trials were classified as low risk of bias, two trials had some concerns, and one trial was classified as high risk of bias owing to deviations from intended interventions. Three trials were open-label and the remaining 8 were double-blind. Risk of bias assessments in individual studies, including reasons, are listed in the characteristics of included studies in Supplementary Table S1.

### 3.3 Primary outcome

#### 3.3.1 Recurrence-free survival

**FIGURE 1:**
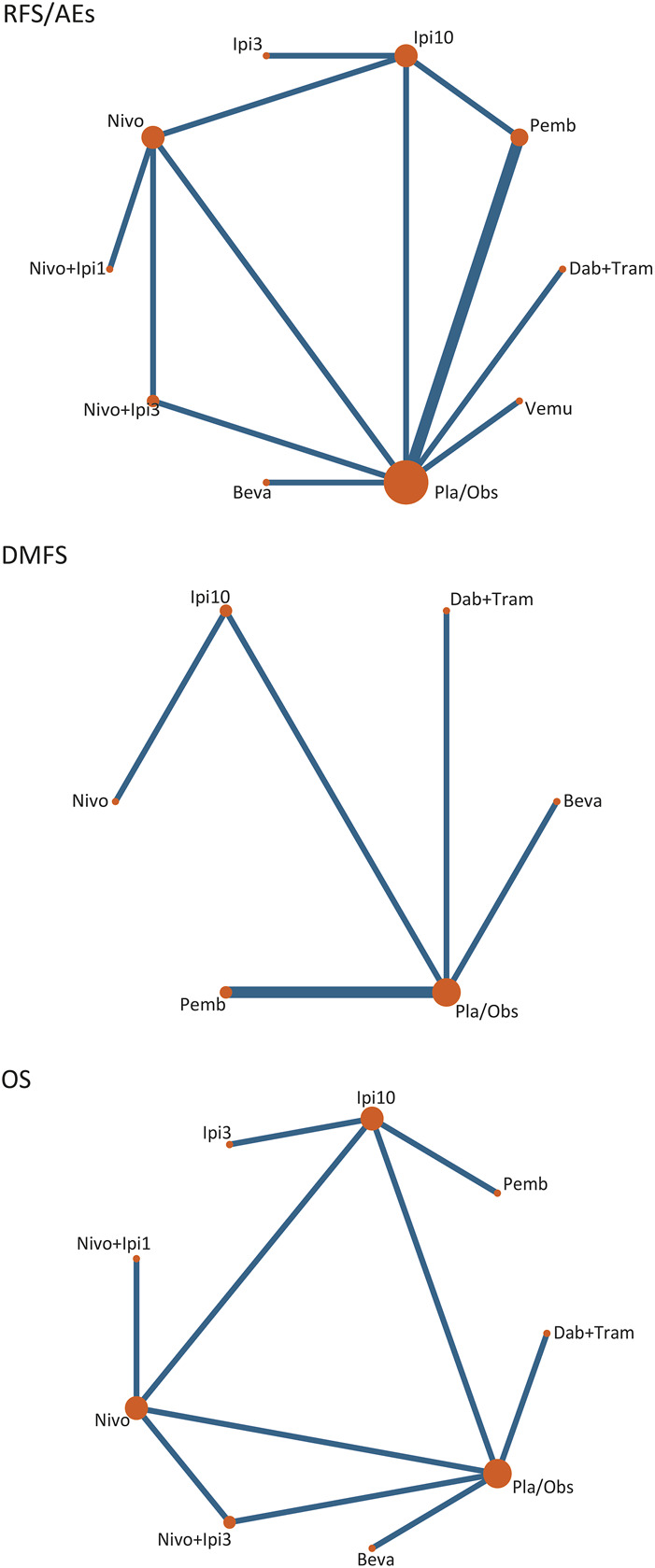
Network plots of treatment comparisons. RFS, recurrence-free survival; OS, overall survival; DMFS, distant metastasis-free survival; Nivo+Ipi3, nivolumab plus ipilimumab 3 mg/kg; Dab+Tram, dabrafenib plus trametinib; Nivo+Ipi1, nivolumab plus ipilimumab 1 mg/kg; Nivo, nivolumab; Pemb, pembrolizumab; Vemu, vemurafenib; Ipi10, ipilimumab 10 mg/kg; Ipi3, ipilimumab 3 mg/kg; Beva, bevacizumab; Pla/Obs, placebo/observation.

**FIGURE 2:**
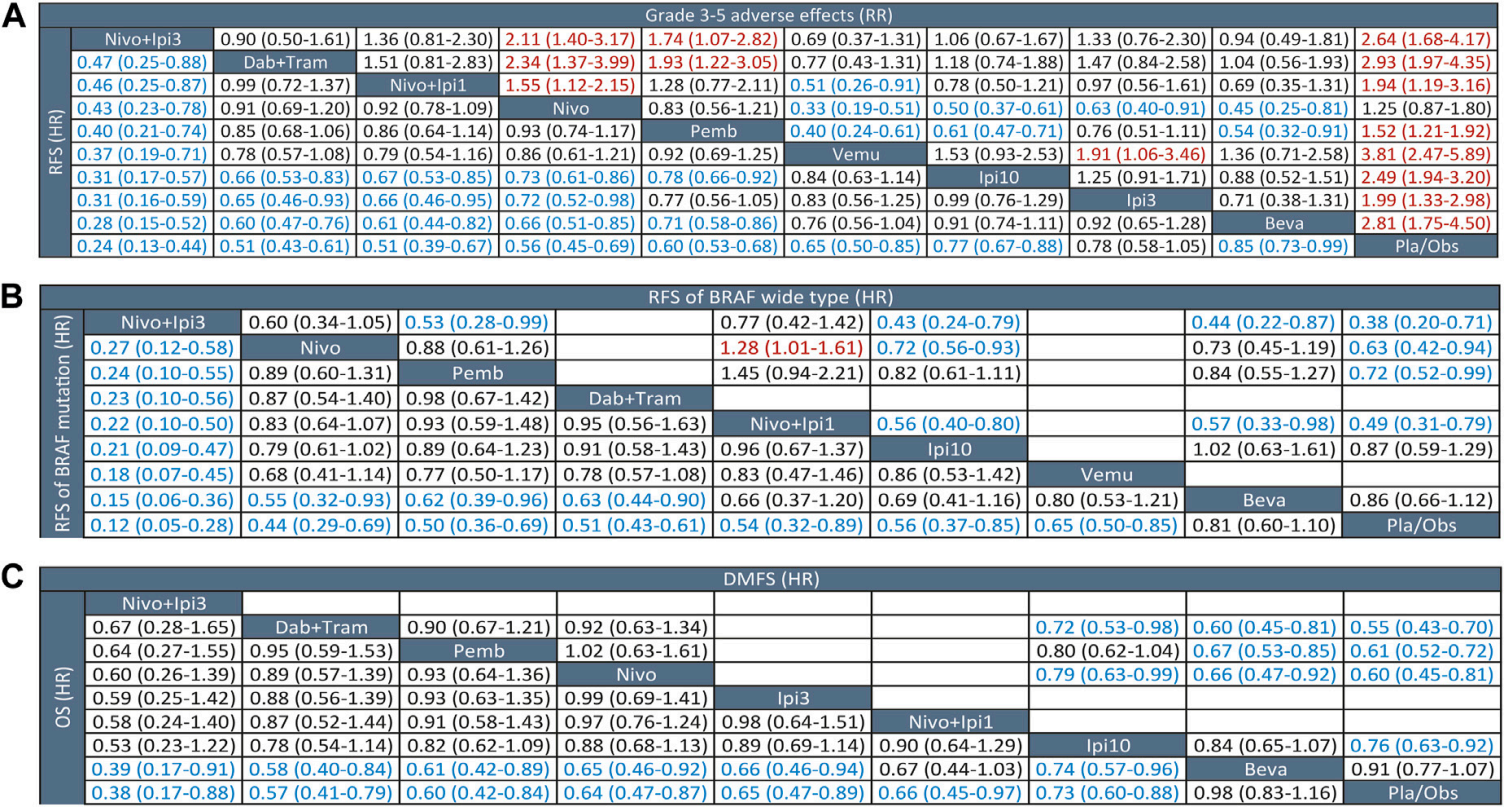
League tables of outcome analyses. **(A)** RFS (lower left) and grade 3-5 adverse effects (upper right). **(B)** RFS of BRAF mutation (lower left) and wide type (upper right). **(C)** OS (lower left) and DMFS (lower left). RFS, recurrence-free survival; OS, overall survival; DMFS, distant metastasis-free survival; Nivo+Ipi3, nivolumab plus ipilimumab 3 mg/kg; Dab+Tram, dabrafenib plus trametinib; Nivo+Ipi1, nivolumab plus ipilimumab 1 mg/kg; Nivo, nivolumab; Pemb, pembrolizumab; Vemu, vemurafenib; Ipi10, ipilimumab 10 mg/kg; Ipi3, ipilimumab 3 mg/kg; Beva, bevacizumab; Pla/Obs, placebo/observation.

Eleven trials (10 treatments) including 10,712 participants reported RFS (Figure 1). In the NMA, we found that all treatments showed benefit over Pla/Obs except Ipi3 (HR, 0.78; 95% CI, 0.58–1.05) (Figure 2A). Treatment ranking probabilities suggested that Nivo + Ipi3 had the highest probability (P-score 0.997) of being the best treatment, which significantly improved RFS compared with other treatments. Compared with Ipi10, RFS benefit was demonstrated for Nivo + Ipi3 (HR, 0.31; 95% CI, 0.17–0.57), Dab + Tram (HR, 0.66; 95% CI, 0.53–0.83), Nivo + Ipi1 (HR, 0.67; 95% CI, 0.53–0.85), Nivo (HR, 0.73; 95% CI, 0.61–0.86), and Pemb (HR, 0.78; 95% CI, 0.66–0.92). There was no significant difference in RFS between Dab + Tram (P-score 0.791), Nivo + Ipi1 (P-score 0.784), Nivo (P-score 0.661), Pemb (P-score 0.569), and Vemu (P-score 0.479). There was no heterogeneity among treatments (I^2^ = 0%). We did not find substantial evidence of inconsistency between direct and indirect evidence.

#### 3.3.2 Subgroup analysis

We performed a prespecified subgroup analysis for BRAF status. Eight trials (9 treatments) were included for patients with BRAF mutations, and NMA showed that all treatments showed benefit over Pla/Obs except Beva (HR, 0.81; 95% CI, 0.60–1.10) (Figure 2B). Nivo + Ipi3 was also the best treatment for mutant BRAF, which significantly improved RFS compared with other treatments. Only six trials (7 treatments) were included for patients with BRAF-wide type, and NMA showed that all treatments showed benefit over Pla/Obs except Beva and Ipi3 (Figure 2B).

### 3.4 Secondary outcomes

#### 3.4.1 Distant metastasis-free survival

Only six trials (6 treatments) reported data on DMFS (Figure 1). All treatments showed DMFS as beneficial over Pla/Obs except Beva (HR, 0.91; 95% CI, 0.77–1.07) (Figure 2C). NMA showed that Dab + Tram (P-score 0.882) ranked first for DMFS, followed by Nivo (P-score 0.767), Pemb (P-score 0.731), Ipi10 (P-score 0.401), Beva (P-score 0.192), and Pla/Obs (P-score 0.026), and there was no significant difference between the top 3 treatments. There was no heterogeneity among treatments (I^2^ = 0%) and no significant inconsistency between direct and indirect evidence.

#### 3.4.2 Overall survival

Eight trials (9 treatments) were included for OS (Figure 1). The results of NMA showed that all treatments showed OS benefit over Pla/Obs except Beva (Figure 2C). NMA also showed that Nivo + Ipi3 (P-score 0.900) ranked the best treatment, followed by Dab + Tram (P-score 0.722), Pemb (P-score 0.681), Nivo (P-score 0.592), Ipi3 (P-score 0.573), Nivo + Ipi1 (P-score 0.534), Ipi10 (P-score 0.357), Beva (P-score 0.085), and Pla/Obs (P-score 0.056), but there were no significant differences between the first 7 treatments. No heterogeneity (I^2^ = 0%) and significant inconsistency was found.

#### 3.4.3 Grade 3-5 adverse events

Eleven trials (10 treatments) including 10,712 participants reported data of grade 3–5 AEs (Figure 1). In the NMA, we found that all treatments were associated with an increased risk of grade 3–5 AEs over Pla/Obs except Nivo (RR, 1.25; 95% CI, 0.87–1.80) (Figure 2A). Treatment ranking probabilities suggested that Nivo (P-score 0.880) and Pemb (P-score 0.763) were the two safest treatments except Pla/Obs. There was no significant difference in AEs between Nivo + Ipi1 (P-score 0.577), Ipi3 (P-score 0.565), Ipi10 (P-score 0.356), Nivo + Ipi3 (P-score 0.307), Beva (P-score 0.270), and Dab + Tram (P-score 0.229). In addition, Vemu (P-score 0.064) ranked the worst treatment. There was moderate heterogeneity among treatments (I^2^ = 63.2%), but no significant inconsistency between direct and indirect evidence was found.

## 4 Discussion

This systematic review and NMA included 11 RCTs (10 treatments) involving 10,712 patients and provided an overview of efficacy and safety outcomes of different treatment strategies in patients with high-risk resected melanoma. We found that the combination therapy of Nivo + Ipi3 ranks highest in terms of RFS and OS, and demonstrates superior RFS compared with other treatments. In addition, Nivo is found to have the lowest risk of grade 3–5 AEs and shows significant benefit compared to other treatment strategies except for Pemb. Meanwhile, both Nivo and Pemb are only inferior to Nivo + Ipi3 in terms of RFS. Although combination therapies including Nivo + Ipi3, Dab + Tram, and Nivo + Ipi1 rank as the top three treatments in terms of RFS, they do not show a significant difference in OS compared to monotherapies including Pemb, Nivo, Ipi3, and Ipi10. Furthermore, Vemu has been found to be the least tolerable treatment with the highest risk of grade 3–5 AEs, and Beva ranks last in terms of RFS, DMFS, and OS.

This is the latest and comprehensive NMA to investigate the efficacy and safety of ICIs and targeted therapy in adjuvant treatment of high-risk resected melanoma depending on evidence from RCTs. This review has several strengths. Firstly, the NMA followed the PRISMA guidelines and had a protocol registered in PROSPERO. We conducted a comprehensive search of multiple databases and included all available RCTs of ICIs and targeted therapy for patients with resected melanoma. Secondly, the risk of bias for included trials was assessed using a valid methodological tool. Thirdly, we have performed a comprehensive analysis including three efficacy outcomes (RFS, DMFS, and OS), subgroups of BRAF status (mutation or wide type), and safety outcome (grade 3–5 AEs) to facilitate optimal evidence-informed decision-making for people and clinicians regarding adjunct treatment strategies for resected melanoma.

One NMA of six trials published in Chinese had previously examined ICIs and targeted therapy for resected melanoma, (Zhang et al., 2022), and its results on RFS were consistent with our review. However, our study included a greater number of trials and treatment strategies and provided more comprehensive insights into DMFS, OS, and safety. Another NMA comparing adjuvant Nivo to other treatments in adults with resected melanoma concluded that Nivo provides an effective treatment option with a promising risk-benefit profile. (Toor et al., 2021). However, the literature search for the previous review was conducted in 2019, and some of the latest RCTs involving ICIs and targeted therapy were not included in the assessment. Furthermore, Ba and colleagues analyzed the efficacy and tolerability of adjuvant therapy for resected high-risk stage III-IV cutaneous melanoma, and the results suggested that Nivo and Pemb seem to be preferable adjuvant therapies. (Ba et al., 2023). However, it should be noted that most of the studies included in the analysis did not involve current ICIs or targeted therapy, and many of the trials were open-label and conducted in the early stages of research.

Although we compared grade 3–5 AEs between different therapies, the most common adverse events were different in these therapies. For example, fatigue and maculopapular rash were the most common events among patients treated with pembrolizumab, while fatigue and diarrhea were most common among patients treated with ipilimumab in the SWOG S1404 trial. In the COMBI-AD trial, comprising dabrafenib plus trametinib, the most common events were pyrexia, fatigue, and nausea.

Although we compared grade 3–5 AEs among different therapies, the most common events varied across these treatments. For instance, among patients treated with pembrolizumab, fatigue, and maculopapular rash were the most common events, whereas fatigue and diarrhea were more prevalent among patients treated with ipilimumab in the SWOG S1404 trial. (Grossmann et al., 2022). In the COMBI-AD trial, the dabrafenib plus trametinib group reported pyrexia, fatigue, and nausea as the most common events. (Long et al., 2017).

In the IMMUNED trial, combination therapy of Nivo + Ipi demonstrated superiority in terms of RFS compared to Nivo monotherapy. (Zimmer et al., 2020; Zimmer et al., 2022). However, in the CheckMate 915 trial, (Weber et al., 2023), this superiority was not observed, which may be due to the lower and less frequent dosage of Ipi used (83% lower exposure). Of note, the IMMUNED trial was the only phase 2 trial included, with only 167 patients enrolled, which is much smaller than other trials. Therefore, future large-scale phase 3 trials of Nivo + Ipi3 in resected melanoma are needed to further investigate its efficacy and safety.

### 4.1 Limitations

This study has several limitations. First, the number of enrolled trials is insufficient, and most of the evidence was derived from indirect comparisons due to the nature of the network analysis. Second, some trials did not report data on DMFS, OS, and BRAF status, which limited our comprehensive analysis of these outcomes due to missing data. Third, moderate heterogeneity was present among the included trials for grade 3–5 AEs. Finally, our analysis was based on study-level data rather than individual patient data, which limited the power of our analysis. Despite these limitations, we believe that our study provides the most comprehensive and up-to-date analysis of ICIs and targeted therapy in the adjuvant treatment of resected melanoma.

## 5 Conclusion

This systematic review and NMA identified 11 RCTs with 10,712 patients with high-risk resected melanoma. This study found that Nivo and Pemb are the preferred options in patients with resected melanoma considering the benefits and harms. Combination therapy of Nivo + Ipi3 showed the most significant improvement in RFS, but the risk of AEs was higher than Nivo and Pemb, highlighting the need for additional evidence from phase 3 trials. Additionally, although combination therapy showed a favorable improvement in RFS compared to monotherapy, this improvement did not translate into an OS benefit based on current evidence. Furthermore, Vemu is not recommended due to its poor tolerability, and Beva is not preferred due to its lower efficacy.

## Data Availability

The original contributions presented in the study are included in the article/Supplementary Material, further inquiries can be directed to the corresponding author.
