# Supplementary material for: Efficacy and safety of immune checkpoint inhibitors and targeted therapies in resected melanoma: a systematic review and network meta-analysis
Source: Front Pharmacol. 2023 Nov 7;14:1284240. doi: 10.3389/fphar.2023.1284240 (PMC10661889; doi:10.3389/fphar.2023.1284240)
Supplement: Supplementary file 1 [file DataSheet1.docx]

Supplementary Material

# Search strategy

**Search strategy for Embase (3475 items)**

1. 'melanoma'/exp
2. melanoma*:ti,ab
3. #1 OR #2
4. adjuvant:ti,ab OR resect*:ti,ab
5. trial*:ti,ab OR random*:ti,ab OR 'controlled clinical trial'/exp
6. #3 AND #4 AND #5

**Search strategy for PubMed (1903 items)**

1. melanoma[MeSH Terms]
2. melanoma*[Title/Abstract]
3. #1 OR #2
4. adjuvant[Title/Abstract] OR resect*[Title/Abstract]
5. Controlled Clinical Trial[MeSH Terms]
6. trial*[Title/Abstract] OR random*[Title/Abstract]
7. #5 OR #6
8. #3 AND #4 AND #7

**Search strategy for Cochrane Library (1219 items)**

1. MeSH descriptor: [melanoma] explode all trees
2. melanoma:ti,ab,kw
3. #1 OR #2
4. adjuvant:ti,ab OR resect*:ti,ab
5. #3 AND #4

**Search strategy for Clinicaltrial.gov (110 items)**

Completed Studies | Studies With Results | melanoma* | adjuvant OR resect*

# Supplementary Figure


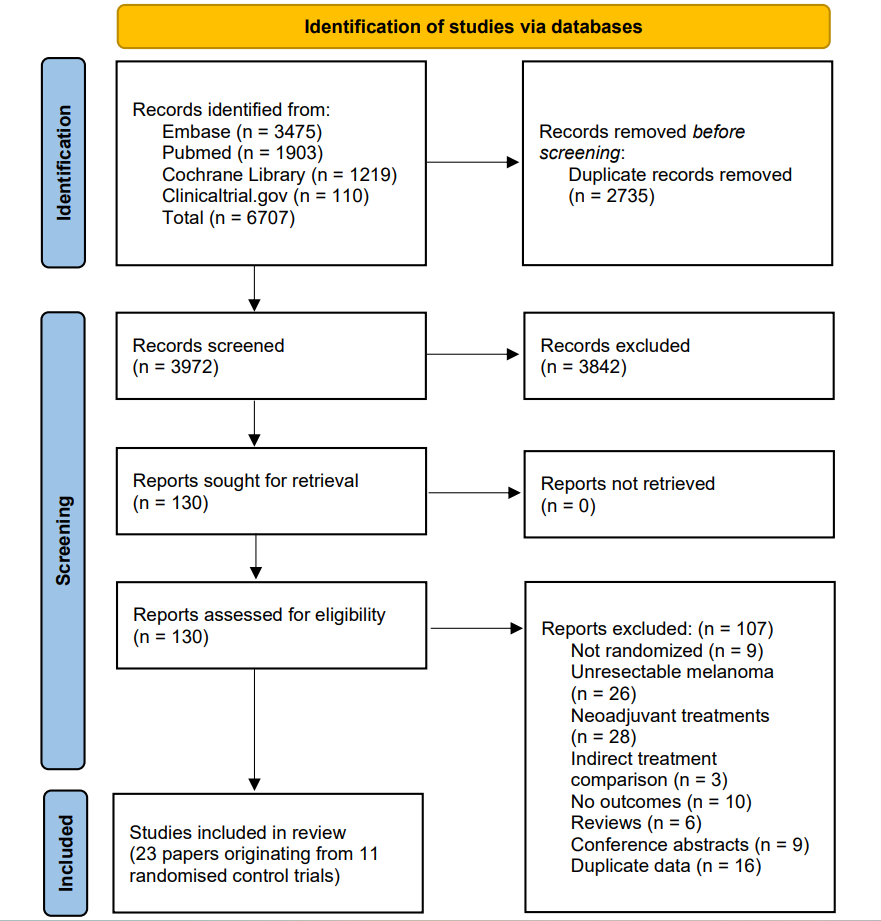


**Supplementary Figure 1.** Study selection

# Supplementary Table

Table S1. Risk of bias for included randomized controlled trials

| **Study** | **Randomization process** | **Deviations from intended interventions** | **Missing outcome data** | **Measurement of the outcome** | **Selection of the reported result** | **Overall bias** |
| --- | --- | --- | --- | --- | --- | --- |
| CheckMate 238 | Low | Low | Low | Low | Low | Low |
| BRIM8 | Low | Low | Low | Low | Low | Low |
| EORTC 1325/KEYNOTE-054 | Low | Low | Low | Low | Low | Low |
| EORTC 18071 | Low | Low | Low | Low | Low | Low |
| AVAST-M | Low | Some concerns^a^ | Low | Low | Low | Some concerns |
| COMBI-AD | Low | Low | Low | Low | Low | Low |
| KEYNOTE-716 | Low | Low | Low | Low | Low | Low |
| SWOG S1404 | Low | High^a,b^ | Low | Low | Low | High |
| IMMUNED | Low | Low | Low | Low | Low | Low |
| CheckMate 915 | Low | Low | Low | Low | Low | Low |
| E1609 | Low | Some concerns^a^ | Low | Low | Low | Some concerns |

^a^ Three trials (AVAST-M, SWOG S1404, and E1609) were open-label trials.

^b^ Study protocol was amended from high-dose interferon alfa-2b to add ipilimumab as a treatment choice.
